# Whole-Genome Resequencing Reveals Genetic Diversity and Selection Signatures in Honghe Cattle

**DOI:** 10.3390/ani16213541

**Published:** 2026-11-09

**Authors:** Yue’e Gao, Yi Jiang, Gexin Chen, Zexuan Hong, Xiaoqian Wu, Jianwei Shi, Jicai Zhang, Xuewei Liu, Jiaxin Chen, Naqash Khan, Chuzhao Lei, Ankui Wang

**Affiliations:** 1 Key Laboratory of Beef Cattle Genetics, Breeding and Reproduction Technology of Yunnan Province, Yunnan Academy of Grassland and Animal Science, Kunming 650000, China; gye11722@126.com (Y.G.); hongzexuan13@163.com (Z.H.); wxx2267464480@163.com (X.W.); 19987841949@163.com (J.S.); ynzjc@126.com (J.Z.); 2 Key Laboratory of Animal Genetics, Breeding and Reproduction of Shaanxi Province, College of Animal Science and Technology, Northwest A&F University, Yangling 712100, China; jiangyi2022@nwafu.edu.cn (Y.J.); nddklxw2020@126.com (X.L.); 19538346608@163.com (J.C.); naqashkhan2025@nwafu.edu.cn (N.K.); 3 College of Animal Science and Technology, Hunan Agricultural University, Changsha 410128, China; e180201@126.com

**Keywords:** Honghe cattle, whole-genome resequencing, genetic diversity, selection signature, Y-chromosome

## Abstract

Honghe cattle were formally recognized as the eighth indigenous cattle breed in Yunnan Province, a region in southwestern China characterized by complex geography and variable climates that have shaped distinctive local cattle germplasm. In this study, we generated whole-genome resequencing data from 62 Honghe cattle and compared them with additional reference samples from other breeds. The results revealed that Honghe cattle are predominantly of indicine origin (54.7% East Asian indicine, 26.5% South Asian indicine and 18.8% taurine), with relatively high genetic diversity. Runs of homozygosity analysis revealed low inbreeding (F_ROH_ = 0.032) and identified 52 ROH islands, the strongest of which (chr7) harbored a protein homeostasis gene cluster (*HSPA9*, *SIL1*, *DNAJC18*). For selection signatures, we applied multiple methods, including composite likelihood ratio test (CLR), integrated haplotype score (*iHS*), fixation index (*F*_ST_) and cross-population extended haplotype homozygosity (XP-EHH) to identify selective sweeps in the Honghe cattle genome. We detected several candidate genes associated with important traits, such as *HMGA2* (body size) and *MCM6* (milk production). Functional enrichment analyses revealed pathways related to immune response, protein absorption, energy metabolism, and lipid metabolism. In addition, Y-chromosomal haplotype analysis revealed that Honghe cattle predominantly belong to the Y3 lineage (Y1 4.5%, Y2 9.1%, Y3 86.4%). These findings inform strategies for improving production efficiency and guiding breeding programs in Honghe cattle.

## Simple Summary

Honghe cattle is formally recognized as the eighth indigenous cattle breed in Yunnan Province, China. In this study, we performed whole-genome resequencing analysis of 62 individuals to investigate their genetic background and identify genes associated with economically important traits. Our results demonstrate that the Honghe cattle population is predominantly of East Asian indicine ancestry, with low levels of inbreeding. Using multiple complementary selection-signature detection methods, we identified several candidate genes associated with heat tolerance, meat quality, immunity, and body size. These findings clarify the unique genetic makeup of Honghe cattle and provide a basis for conserving and improving this valuable local breed.

## 1. Introduction

Domestic cattle are broadly classified into taurine (*Bos taurus*) and indicine (*Bos indicus*), which diverged approximately 200,000–300,000 years ago from two primary domestication centres in the Near East and the Indus Valley [1]. Taurine cattle are adapted to temperate and cold climates and are distributed predominantly across the Northern Hemisphere [2]. In contrast, indicine cattle exhibit strong heat and drought tolerance, making them suited to tropical and subtropical climates, and are distributed mainly across equatorial regions and the Southern Hemisphere, particularly in South Asia, Southeast Asia, southern East Asia, and Africa [3,4]. Crossbred offspring of taurine and indicine cattle, which exhibit heterosis and complementary traits, are distributed primarily in regions where temperate and tropical climates converge [5,6]. Chinese indigenous cattle constitute a major component of global *Bos* genetic resources, comprising 58 recognized local breeds [7], These breeds derive from five ancestral components: Eurasian taurine, European taurine and East Asian taurine in northern China, East Asian indicine and South Asian indicine cattle in southern China [8,9].

Yunnan Province possesses rich cattle genetic diversity, with eight local breeds documented as of 2025 [7]. Its geographical position borders South Asia and Southeast Asia, creating a contact zone for indicine and taurine cattle. Consequently, cattle populations in Yunnan harbor mixed taurine and indicine ancestry [8], comprising predominantly East Asian indicine and South Asian indicine lineages, with South Asian indicine ancestry increasing gradually from northern to southern Yunnan [10]. Moreover, under the region’s distinct environmental conditions, these cattle populations have evolved heat tolerance and disease resistance adaptive to local environment.

Honghe cattle are distributed widely across Honghe Prefecture, located along the China–Vietnam border in southern Yunnan Province. They are reared primarily under grazing systems and are classified as a local dual-purpose breed for meat and draft use, representing the smallest-bodied local cattle breed in Yunnan. Most individuals exhibit a prominent hump, with relatively small dewlaps and thoracic folds. Owing to their strong capacity for steep-slope climbing, tolerance of high temperatures and humidity, resistance to drought and ability to utilize roughage, Honghe cattle occupy an important position among the genetic resources of Yunnan Province [11]. However, Honghe cattle farming is currently facing multiple pressures, including grassland degradation, population decline, outdated breeding techniques, and limited market access [12]. Consequently, continued efforts are needed to improve breeding practices and refine breeding strategies for this population using scientific approaches.

Genetic variation in livestock arises from both natural selection and artificial selection. Recently, whole-genome sequencing has been widely used to characterize livestock population structure, genetic diversity, and selection signatures [13,14]. However, the genetic background, ancestral origins, and selection signatures of Honghe cattle remain uncharacterized at the whole-genome level.

To address this gap, we performed whole-genome sequencing of 62 individuals and identified single nucleotide polymorphisms (SNPs) based on the bovine reference genome (ARS-UCD1.2). This study aimed to characterize the genomic characteristics and inbreeding levels underlying the diversity of Honghe cattle using the largest genomic dataset of this breed assembled to date. By investigating population structure, genomic variation, and selective sweep signals, we aimed to identify distinctive environmental adaptation features of this breed. These findings will advance understanding the genomic architecture of this breed and identifying candidate genomic regions of interest. Moreover, it will serve as a reference for genomic studies of other indigenous cattle breeds and advance the research on local breeds in Yunnan Province.

## 2. Materials and Methods

### 2.1. Sample Collection and Genome Sequencing

**Figure 1:**
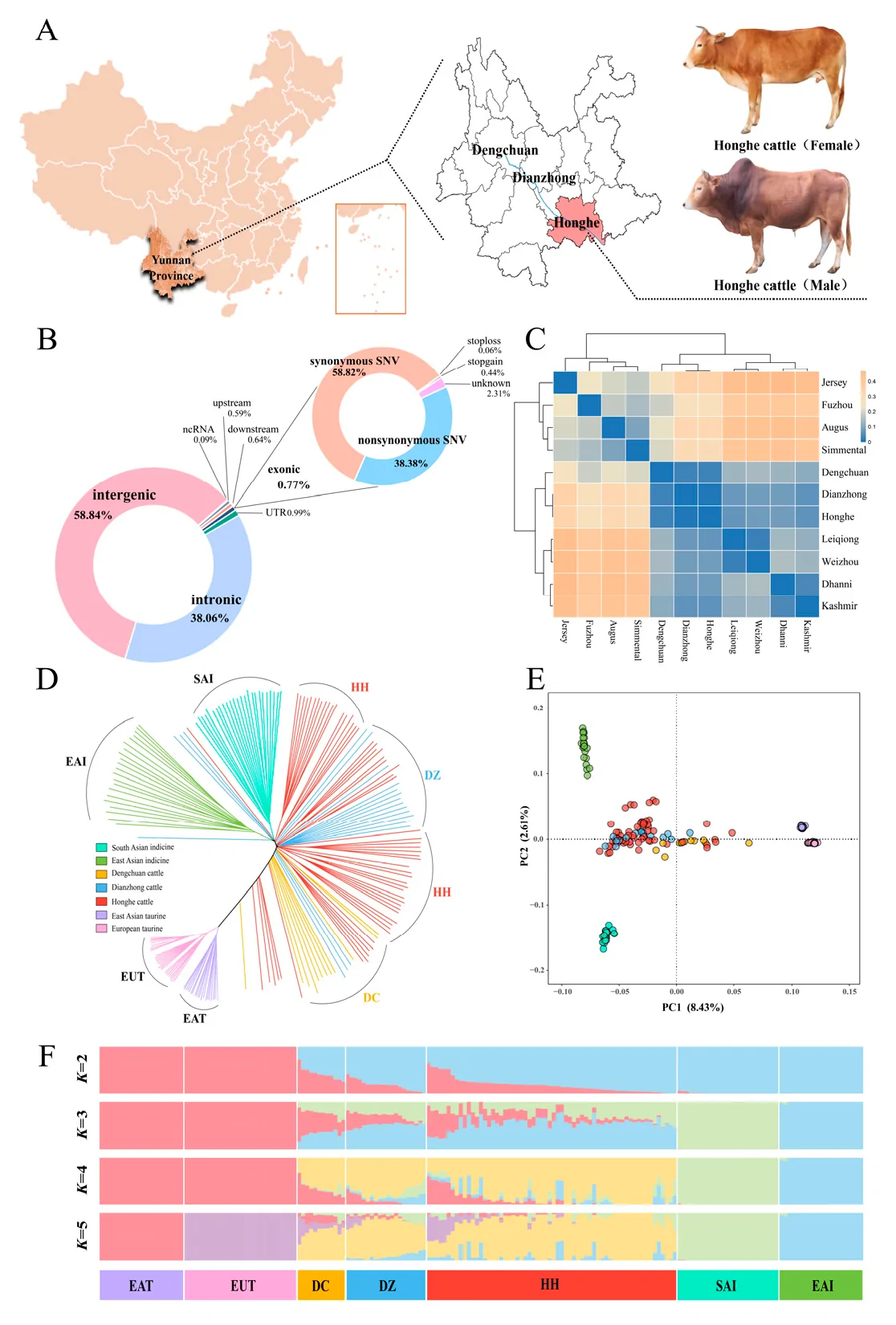
Population genetic structure of Honghe cattle. (**A**) Geographic distribution of Honghe cattle. (**B**) Functional classification of the detected SNPs. (**C**) Population differentiation index (*F*_ST_) among populations. (**D**) Neighbor-joining tree of the relationships in these populations. (**E**) Principal component analysis of cattle with PC1 against PC2. (**F**) Ancestry component analysis of these cattle breeds us-ing ADMIXTURE with *K* = 2 and *K* = 5 (For each *K*, each color represents a distinct ancestral component).

We collected blood samples from 62 Honghe cattle in Honghe Prefecture, Yunnan Province (Figure 1A). Blood collection was performed by professional veterinarians, and the collection sites were disinfected immediately afterward in accordance with animal welfare guidelines. Genomic DNA was extracted from the blood samples using the standard phenol–chloroform method [15]. After determining DNA concentration and quality, we constructed paired-end libraries using the Illumina NovaSeq 6000 platform (Illumina, Beijing, China), with an average insert size of 500 bp and a read length of 150 bp for each individual. Sequencing was performed by Novogene Bioinformatics Research Institute in Beijing, China. The sequencing data have been deposited in the National Genomics Data Center (NGDC, https://ngdc.cncb.ac.cn, accessed on 5 November 2026) under the BioProject accession number PRJCA072462. To provide comparative genomic context, we additionally retrieved raw whole-genome sequencing data (FASTQ format) from 127 publicly available samples spanning five major lineages: Yunnan local breeds geographically close to Honghe cattle (Dianzhong, *n* = 20; Dengchuan, *n* = 12; Figure 1A), South Asian indicine (Kashmir, *n* = 20; Dhanni, *n* = 5), East Asian indicine (Leiqiong, *n* = 4; Weizhou, *n* = 17), European taurine (Angus, *n* = 10; Simmental, *n* = 8; Jersey, *n* = 10), and East Asian taurine (Fuzhou, *n* = 21). In total, 189 samples were used in this study (Table S1).

### 2.2. Read Mapping and SNP Calling

Raw sequencing data were first subjected to quality control, during which low-quality reads and adapter sequences were removed using Trimmomatic (version 0.39) [16]. Clean reads were then aligned to the bovine reference genome (ARS-UCD1.2) using the BWA-MEM (version 0.7.17-r1126) [17]. The resulting BAM files were sorted by chromosome and duplicate reads were removed using Picard tools (http://broadinstitute.github.io/picard, accessed on 5 November 2026). SNP calling and filtering were performed using the HaplotypeCaller, GenotypeGVCFs, SelectVariants and VariantFiltration modules of GATK (version 3.8), applying the following filtering criteria: (1) mean sequencing depth for all samples (*n* = 189) >1/3× and <3×; (2) QD < 2; (3) FS > 60.0; (4) MQ < 40; (5) MQRankSum < −12.5; (6) ReadPosRankSum < −8; (7) SOR > 3.0. Threshold selection followed GATK best practices and previous bovine genomic studies [18]. Variants with a depth of coverage <1/3× or >3× of the mean depth were excluded. Finally, the high-quality SNPs were annotated using ANNOVAR for subsequent analyses [19].

### 2.3. Population Structure and Phylogenetic Analysis

For population genetic analyses, PLINK (version 1.9) was used to prune SNPs in linkage disequilibrium using the parameter --indep-pairwise 50 5 0.2 [20]. VCFtools (version 0.1.17) was employed for file format conversion [21]. Principal component analysis (PCA) and fixation index (*F*_ST_) calculations were performed using the smartpca module in the EIGENSOFT (version 5.0) [22]. Genetic structure of the 189-sample dataset was inferred using ADMIXTURE (version 1.3) [23], with the number of ancestral populations (*K*) set from 2 to 6. Cross-validation was performed for each *K* value, and the optimal *K* was determined based on the lowest cross-validation error. Phylogenetic analysis was conducted using PLINK to construct a pairwise genetic distance matrix via the neighbor-joining (NJ) method, with visualization performed using MEGA-X (version 10.0) [24] and the iTOL website (https://itol.embl.de, accessed on 5 November 2026) [25].

### 2.4. Genomic Diversity, Inbreeding and ROH Detection

Basic SNP statistics (total number of SNPs and breed-specific SNPs) were calculated using BCFtools (version 1.9) [26]. Nucleotide diversity (π) for each breed was estimated using VCFtools with a window size of 50 kb and a step size of 20 kb [21]. Runs of homozygosity (ROH) were identified within each breed separately using BCFtools with genotype calls only (-GTs-only 30), and segments were filtered by three criteria including length ≥ 500 kb, ≥100 SNPs and HMM quality score >50 [27]. The BCFtools/RoH hidden Markov model was run under default parameters, assuming a constant recombination rate (1 cM ≈ 1 Mb), with allele frequencies estimated from the sample itself.

The inbreeding coefficient was calculated as F_ROH_ = total ROH length/autosomal genome length (2489.39 Mb, sum of chromosomes 1–29 in ARS-UCD1.2), individuals without ROHs were assigned F_ROH_ = 0. Following common classifications in ROH studies [14,28,29], ROHs were grouped into four length classes: (1) 0.5–1 Mb, (2) 1–2 Mb, (3) 2–4 Mb, and (4) >4 Mb. Based on the filtered segments, the proportion of individuals carrying ≥1 ROH overlapping each 50 kb window (step 50 kb) was computed, and windows ranking in the top 1% genome-wide were merged into ROH islands. Genes overlapping the islands were annotated using BEDTools (version 2.31.0) intersect for GO/KEGG analysis [30].

### 2.5. Estimates of the Effective Population Size

We inferred the recent demographic history of each population using SMC++ (version 1.15.5) [31]. Autosomal SNPs were called with GATK (version 3.8) using the criteria described above and phased with Beagle (version 4.1) [32]. Only autosomes were analyzed; regions flagged in the reference-genome mask were excluded. Phased VCFs were converted to SMC++ format chromosome by chromosome using smc++ vcf2smc, with each population specified as a single group. Effective population size (*Ne*) trajectories were estimated independently per population with smc++ estimate, assuming a mutation rate of 1.26 × 10^−8^ per site per generation and a generation time of 6 years.

### 2.6. Selective Sweep Detection Within Honghe Cattle

For the detection of selection signatures within the Honghe cattle population, we employed two complementary methods. First, we applied the composite likelihood ratio test (CLR) using SweepFinder2 on whole-genome SNP data [33], assuming a folded site-frequency spectrum estimated empirically from the data and thereby implicitly accounting for demographic history. The maximum CLR per 50 kb window (20 kb step) was retained to identify deviations from the neutral site frequency spectrum that may indicate potential selective sweeps. Second, after phasing and imputing the genotype data using Beagle [32], we computed the integrated haplotype score (*iHS*) using Selscan (version 2.0) [34] on variants with MAF ≥ 0.05. Because no outgroup was available, the ALT allele was treated as derived, and all downstream analyses used |*iHS*|, which is invariant to ancestral/derived labeling. Raw scores were normalized within 100 derived-allele-frequency bins [35], and empirical thresholds were applied to the normalized scores with Benjamini–Hochberg FDR correction. Candidate genes were defined as those intersecting the top 0.5% of signals identified by both methods. Kyoto Encyclopedia of Genes and Genomes (KEGG), Gene Ontology (GO) enrichment analyses of the candidate genes were performed using KOBAS (version 3.0) (http://bioinfo.org/kobas, accessed on 5 November 2026) [36], and pathways with a corrected *p*-value < 0.05 were considered significantly enriched.

### 2.7. Cross-Population Selection Analysis

To identify genomic regions underlying environmental adaptation in Honghe cattle, pairwise *F*_ST_ values were calculated using VCFtools with a 50 kb window size and a 20 kb step size [21]. The cross-population extended haplotype homozygosity (XP-EHH) statistic was calculated to compare Honghe cattle with East Asian taurine, using the same window size (50 kb) and step size (20 kb). The top 0.5% of signals were retained for further analysis. Genes detected by both methods were considered as candidate genes. Haplotype block structure was visualized using LDBlockShow (version 1.40) [37].

### 2.8. Paternal Lineage Analysis

For Y-chromosome analysis, we examined the X-degenerate region within the male-specific region of the Btau_5.0.1 Y-chromosome reference sequence (GCF_000003205.7); the analysis was restricted to two single-copy intervals (Y: 2,526,180–3,977,988 and Y: 42,200,142–43,298,054) to exclude the pseudoautosomal regions and X–Y gametologous regions. The analysis included 22 Honghe bulls and 10 reference individuals. SNPs were called with GATK (version 3.8) as described above, retaining monomorphic sites (-includeNonVariantSites), and filtered to biallelic sites, yielding 2870 variant sites.

Haplotype diversity (Hd ± SD) was calculated using DnaSP (version 6) [38]. Nucleotide diversity (π) was calculated as the mean number of pairwise differences divided by the total surveyed length of the X-degenerate region (L = 2,549,722 bp), with monomorphic sites included. It should be noted that the 659 sites used for phylogenetic reconstruction and the median network analysis represent the final set of positions after complete deletion of sites with missing data or gaps. Based on FASTA-format sequences, the haplogroup tree was constructed using the maximum likelihood (ML) method in MEGA-X [24] under the best-fit Tamura 3-parameter (T92) model selected by BIC, with 1000 bootstrap replicates; all nodes received bootstrap support ≥99%. Finally, a haplotype network was generated based on Y-SNPs using Network (version 10.2) software [39] to visualize relationships among haplotype groups.

## 3. Results

### 3.1. Whole-Genome Sequencing and Variant Detection

Whole-genome resequencing was performed on blood samples from 62 Honghe cattle, achieving an average sequencing depth of 11.3×, a mean mapping rate of 99.78% against the reference genome, and an average duplication rate of 5.95%. Variant calling and annotation identified a total of 47,077,437 single nucleotide polymorphisms (SNPs). The annotation results showed that most SNPs were located in intergenic (58.84%) and intronic (38.06%) regions, with the remainder in exonic regions (0.77%), untranslated regions (0.99%), upstream/downstream and non-coding RNA regions (1.34% combined). Within exonic regions, we identified 213,738 synonymous and 139,450 nonsynonymous variants (Figure 1B; Table S2).

### 3.2. Population Structure and Ancestral Relationships

Principal component analysis (PCA) showed that the first three principal components cumulatively explained 13.3% of the total variance. One-way ANOVA with population as the grouping factor indicated that each of the three axes significantly separated populations, each capturing more than 94% of the among-population variance. Specifically, PC1 (8.42% of total variance) clearly separated taurine from indicine cattle, and PC2 (2.61%) distinguished South Asian from East Asian indicine. The Yunnan local breeds, including Honghe cattle, clustered closely with indicine populations and were positioned between the taurine and indicine clusters, although a few individuals were positioned near taurine populations (Figure 1E and Figures S2–S4; Table S3).

In the *F*_ST_ analysis, pronounced differentiation was observed between taurine and indicine cattle. The three Yunnan breeds showed moderate differentiation from South Asian indicine (0.060–0.107) (Table S3). Notably, the *F*_ST_ value between Honghe cattle and Dianzhong cattle was only 0.008, indicating a close genetic relationship, a result further supported by hierarchical clustering analysis (Figure 1C; Table S4). ADMIXTURE analysis showed that at *K* = 2, Honghe cattle were predominantly of indicine origin. At *K* = 3, the indicine ancestry was partitioned into South Asian and East Asian components, with Honghe cattle comprising 54.70% East Asian indicine, 26.47% South Asian indicine, and 18.83% taurine ancestry. At *K* = 4, Honghe cattle and the other two Yunnan local breeds formed a distinct cluster separated from the reference populations, representing the recent shared population history of the three Yunnan breeds, whose genetic structure is highly distinctive. At *K* = 5 (lowest cross-validation error, Figure S1), the distinctive genetic structure of the three Yunnan breeds was retained, while the ancestry of the seven populations was resolved more clearly (Figure 1F). The neighbor-joining (NJ) tree further supported this complex genetic composition of Honghe cattle (Figure 1D).

### 3.3. Patterns of Genomic Variation

**Figure 2:**
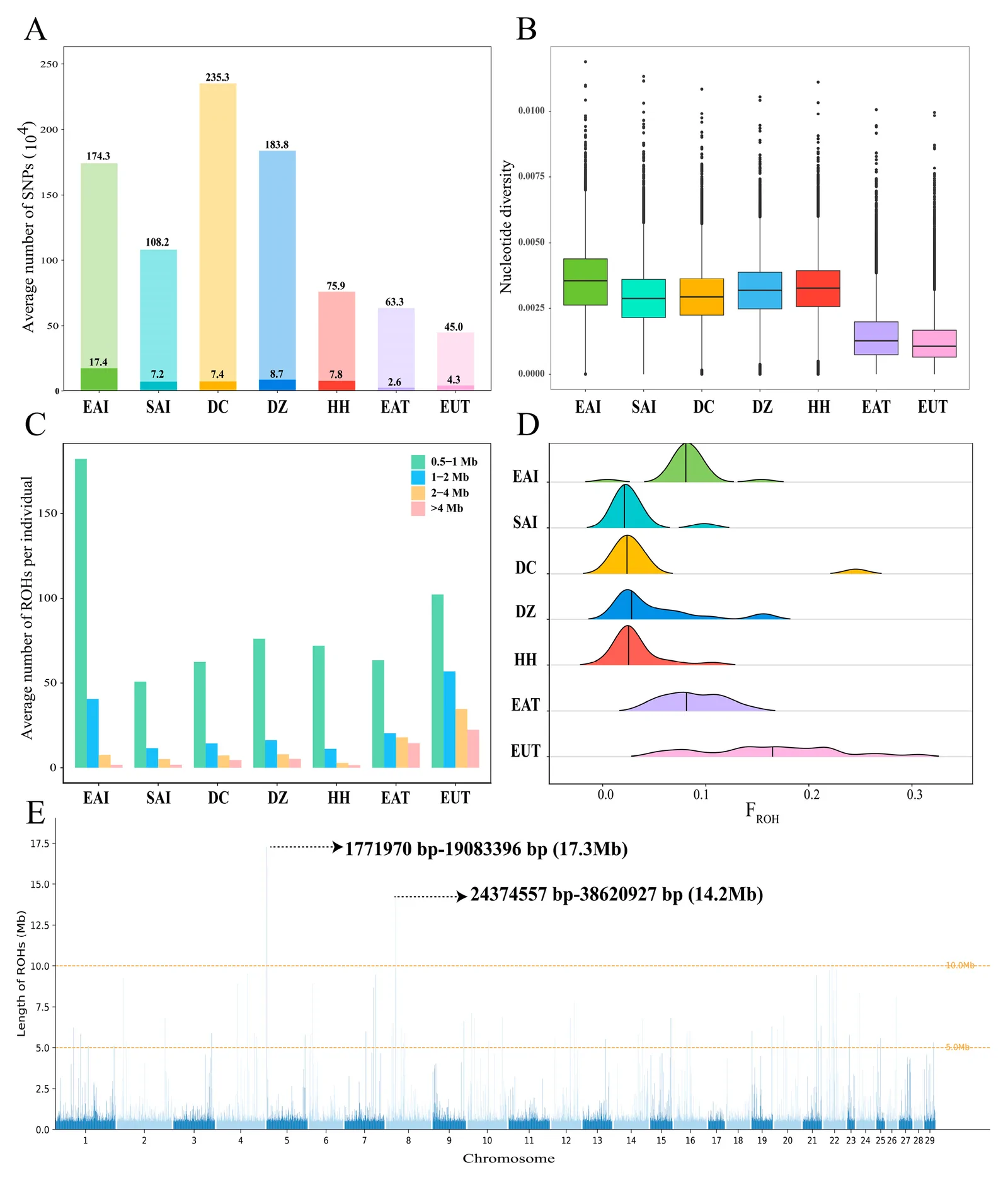
Genomic diversity of Honghe cattle. (**A**) Number of private SNPs in each population (high and low bars represent the numbers of all SNPs and breed-specific SNPs, respectively). (**B**) The genome-wide distribution of nucleotide diversity in each population. (**C**) Average number of four types of ROH in each population. (**D**) F_ROH_ of each population. (**E**) Genomic distribution of ROH in Honghe cattle.

The average number of breed-specific SNPs per individual was highest in East Asian indicine (173.7 k) and much lower in all other populations (26.1 k–87.2 k), with the three Yunnan breeds intermediate (DZ: 87.2 k; HH: 78.4 k; DC: 74.3 k) and the two taurine populations lowest (EUT: 43.3 k; EAT: 26.1 k) (Figure 2A; Table S5). Nucleotide diversity followed a largely consistent trend with SNP counts, with East Asian indicine showing the highest value (0.00355), followed by Honghe cattle (0.00327), and taurine populations the lowest (EAT: 0.00127; EUT: 0.00107) (Figure 2B).

The ROH and F_ROH_ results further supported the patterns observed for SNP numbers and nucleotide diversity. European taurine exhibited substantially higher ROH counts and total lengths than other breeds (mean of 216 segments ≥0.5 Mb per individual, totaling 400.7 Mb), whereas South Asian indicine and Honghe cattle showed the lowest values (73.0 and 78.5 Mb per individual) (Figure 2C and Figure S5; Table S6). Consistently, the median F_ROH_ was highest in European taurine (0.165) and East Asian taurine (0.081), intermediate in East Asian indicine (0.081), and lowest in the three Yunnan breeds and South Asian indicine (HH: 0.025; DC: 0.024; DZ: 0.028; SAI: 0.021) (Figure 2D).

### 3.4. ROH Distribution Characteristics

A total of 5416 high-quality ROH segments (≥ 500 kb) were identified in Honghe cattle, with a cumulative length of 4868.37 Mb, including 96 segments longer than 4 Mb and 2 longer than 12 Mb (Figure 2E; Table S7). Gene annotation of the 96 long ROH segments yielded 5167 genes, and 2682 genes remained after removing LOC and tRNA genes. Enrichment analysis revealed nine significantly enriched KEGG pathways (corrected *p* < 0.05, Figure S6), predominantly related to energy-metabolic homeostasis (metabolic pathways, insulin resistance and adrenergic signaling in cardiomyocytes), cell proliferation and growth (MAPK, Rap1, focal adhesion and pathways in cancer), and nutrient absorption (protein digestion and absorption, driven by a cluster of pepsinogen-homologous PAG/PGA5 proteins)—processes underpinning body development, muscle growth and environmental (heat) adaptation in livestock. Metabolic pathways showed the highest enrichment level (corrected *p* = 1.30 × 10^−8^) (Table S8).

**Figure 3:**
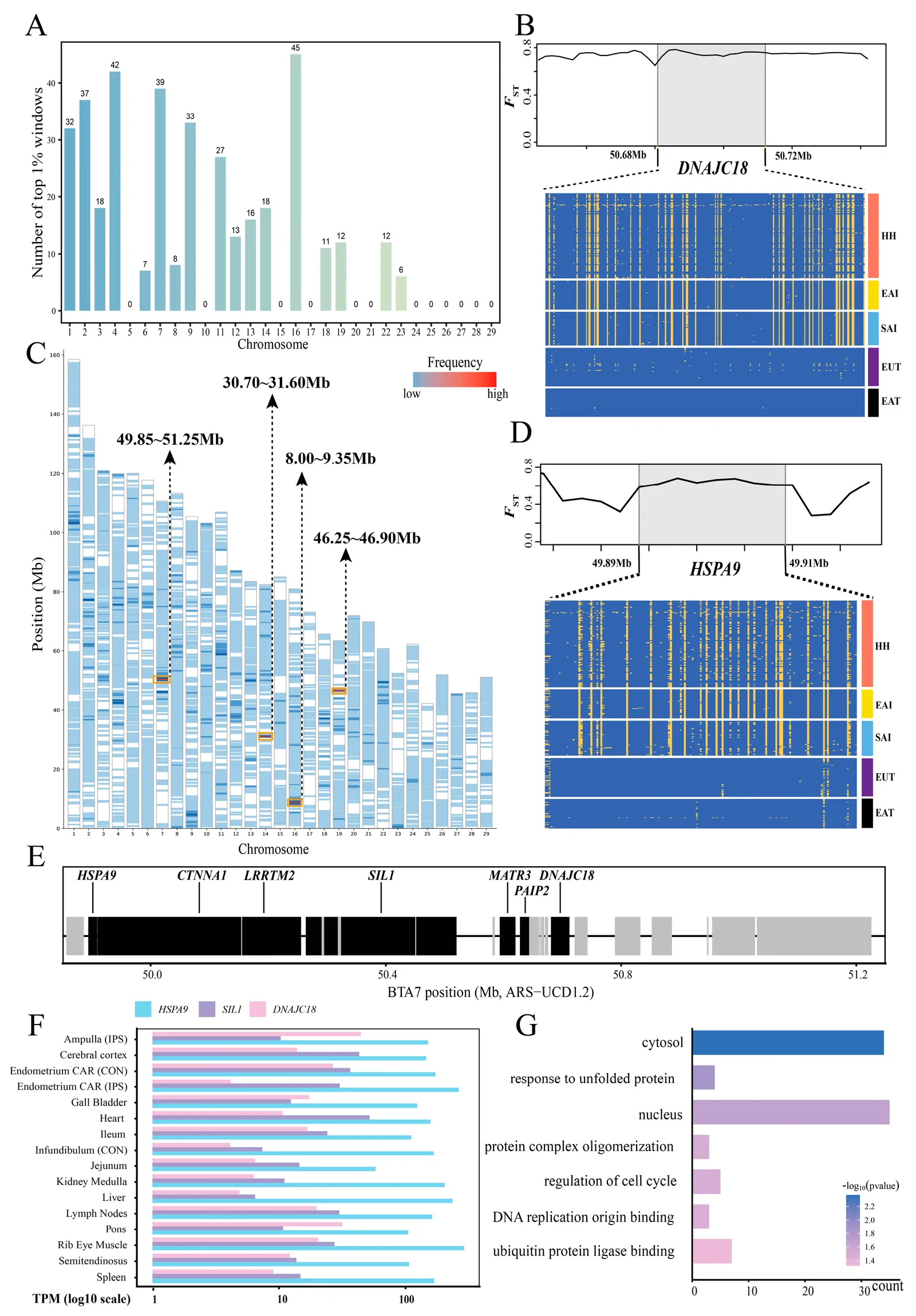
ROH-enriched region analysis in Honghe cattle based on 50 kb sliding windows. (**A**) Chromosomal distribution of the number of top 1% windows. (**B**) Pairwise *F*_ST_, haplotype patterns heatmap of *HSPA9* gene region. (**C**) Genome-wide distribution of per-window ROH frequency across 29 autosomes. (**D**) Pairwise *F*_ST_, haplotype patterns heatmap of *DNAJC18* gene region. (**E**) Gene map of the chr7 island (black boxes denote genes discussed in the text). (**F**) Tissue expression profiles (TPM) of the three protein-homeostasis genes *HSPA9*, *SIL1* and *DNAJC18* based on public RNA-seq data (PRJNA294306). (**G**) Bar graph of GO-enriched terms, colored by *p*-values (corrected *p* < 0.05).

In addition, 52 ROH islands were identified using a 50 kb sliding-window approach (frequency ranked in the top 1% genome-wide) (Figure 3C). Gene annotation of these islands yielded 294 valid genes (excluding uncharacterized LOC and tRNA genes; Table S9). GO enrichment analysis revealed significant terms related to protein homeostasis and cellular quality control, including cytosol (corrected *p* = 0.004), response to unfolded protein (corrected *p* = 0.013) and ubiquitin protein ligase binding, as well as nucleus, regulation of cell cycle and DNA replication origin binding (Figure 3G; Table S11). KEGG analysis showed nominal enrichment (corrected *p* < 0.1) in protein processing in endoplasmic reticulum and ubiquitin-mediated proteolysis (Table S10).

Among the four strongest islands (chr7: 49.85–51.25 Mb, chr14: 30.70–31.60 Mb, chr16: 8.00–9.35 Mb, chr19: 46.25–46.90 Mb), the chr7 island contained a protein-homeostasis gene cluster: *HSPA9*, *SIL1* and *DNAJC18*—together with *CTNNA1*, *LRRTM2*, *MATR3*, *PAIP2* and others; these genes are differentiated in indicine cattle (Figures S8–S12), and their functions are consistent with the GO enrichment results (Figure 3E; Table S9). Public expression data from NCBI (PRJNA294306) showed complementary tissue profiles of the three chaperones: *HSPA9* was maximized in metabolically demanding tissues and gravid endometrium, *SIL1* in heart and brain, and *DNAJC18* in the oviductal ampulla (Figure 3F). Notably, *DNAJC18*, *HSPA9* and related genes exhibited elevated *F*_ST_ values and haplotype patterns distinct from taurine cattle, potentially associated with environmental adaptation in Honghe cattle (Figure 3B,D). In addition, the other strong islands carried functional candidate genes: the chr14 island contained *MYL4* (muscle development) and *SGK3* (hair-follicle-related).

### 3.5. Selective Sweep Detection

**Figure 4:**
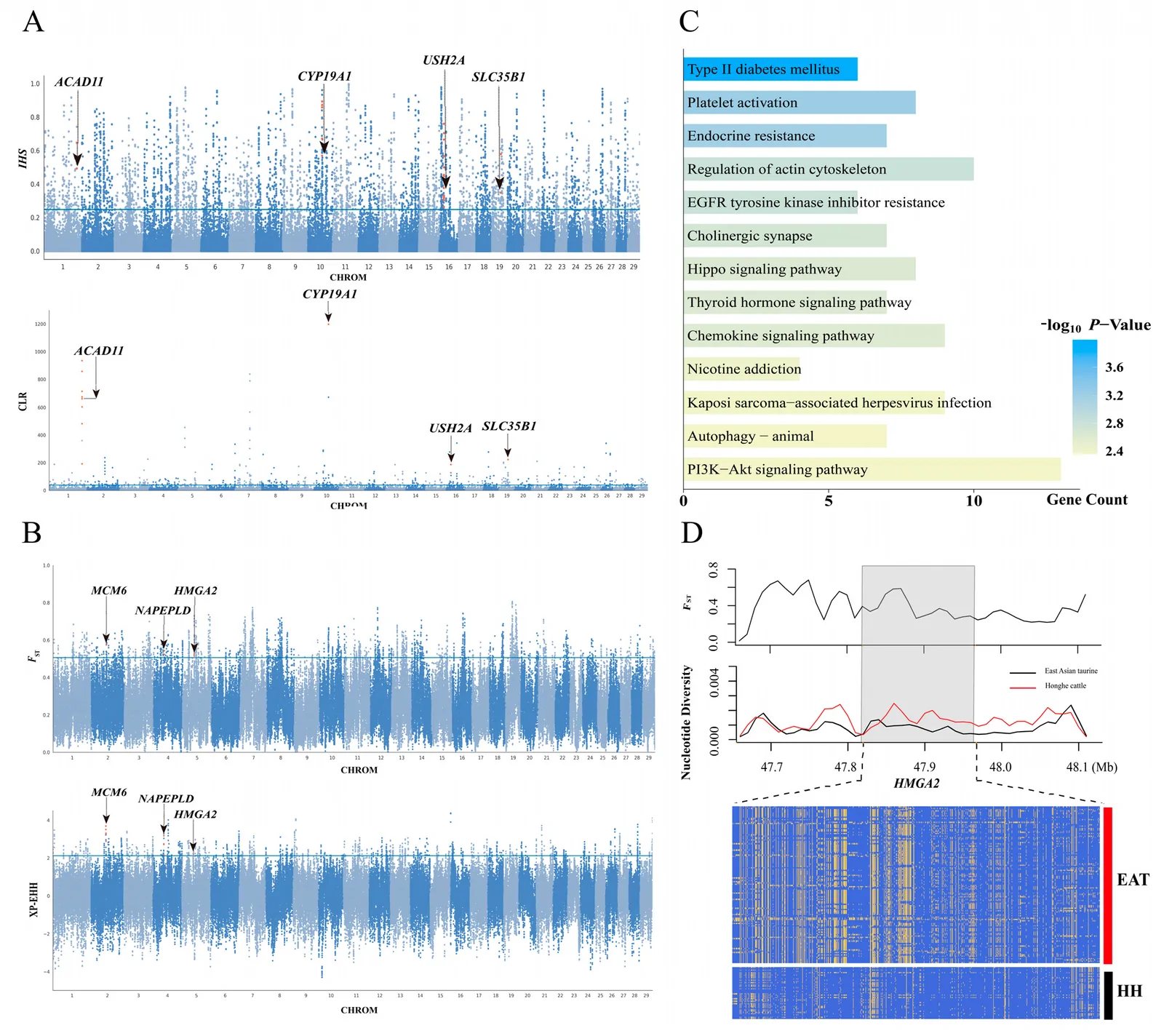
Analysis of environmentally adaptive genes in Honghe cattle. (**A**) Manhattan plots of *iHS* (upper) and CLR (lower) selective sweep scans. (**B**) *F*_ST_ (upper) and XP-EHH (lower) between Honghe cattle and East Asian taurine. (**C**) Bar graph of KEGG-enriched terms, colored by *p*-values (corrected *p* < 0.05). (**D**) Pairwise *F*_ST_, nucleotide diversity, haplotype patterns heatmap of *HMGA2* gene region.

To detect selection signatures within the Honghe cattle population, we applied both CLR and *iHS* methods. A total of 618 genes (*iHS*) and 954 genes (CLR) were annotated, respectively (Figure 4A; Tables S12 and S13). The 95 overlapping genes identified by both methods were considered as candidate genes under positive selection in the Honghe cattle (Figure S13). Among these candidate genes, we identified *CYP19A1* (chromosome 10, reproductive traits), *USH2A* (chromosome 16, coat color), *SLC35B1* (chromosome 19, lactose metabolism), and *ACAD11* (chromosome 1, feed intake variation). These genes are functionally involved in energy metabolism, growth regulation, and physiological homeostasis, processes relevant to meat quality, immunity, body size, and heat tolerance.

To mine loci underlying environmental adaptation in Honghe cattle, we performed cross-population comparisons using the *F*_ST_ and XP-EHH methods and identified 443 overlapping genes (Figure S14; Tables S14 and S15). KEGG enrichment analysis of these 443 genes revealed several significantly enriched pathways, including MicroRNAs in cancer, the chemokine signaling pathway, and the Hippo signaling pathway, which are involved in immune regulation, cell proliferation, and organismal growth and development (Figure 4C; Table S16). Among these genes, we identified *MCM6* (chromosome 2, milk production), *NAPEPLD* (chromosome 4, meat quality), and *HMGA2* (chromosome 5, body size) (Figure 4B). Notably, the *HMGA2* gene exhibited high *F*_ST_ values and a distinctive haplotype pattern, suggesting that this gene may be associated with environmental adaptation in Honghe cattle (Figure 4D).

### 3.6. Y-Chromosome Analysis

**Figure 5:**
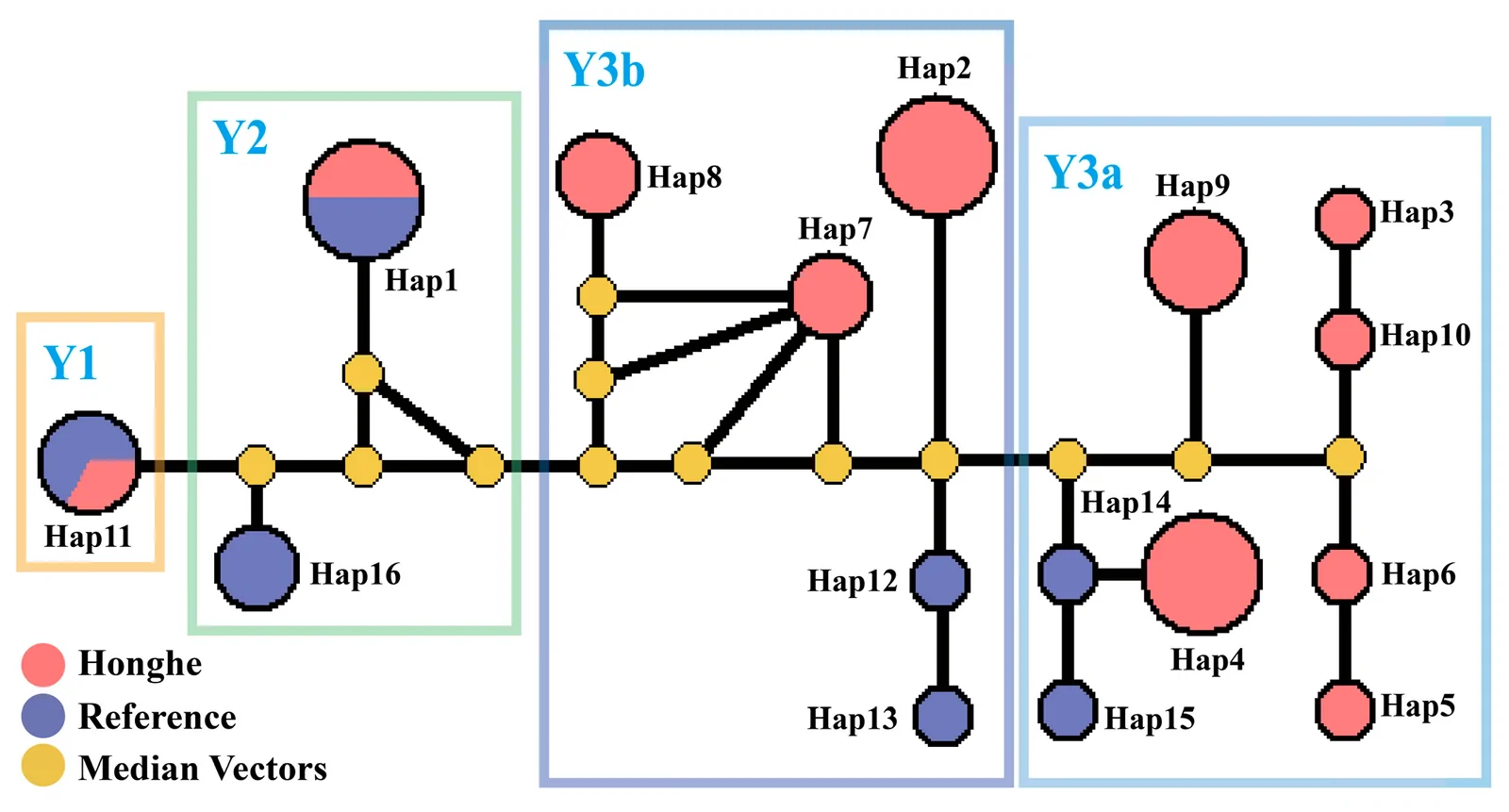
Y-chromosome phylogenetic tree (circle areas are proportional to sample size). Y2 encompasses Y2a and Y2b, and the blue boxes of different depths distinguish Y3a from Y3b.The green and yellow boxes indicate the Y2 and Y1 haplogroups, respectively. A total of 659 effective sites were analyzed, including 419 segregating (polymorphic) sites, with a total of 493 mutations (Eta).

Y-chromosome haplotype analysis of 32 bulls (22 Honghe cattle and 10 reference individuals) defined 16 distinct haplotypes (haplotype diversity Hd = 0.946 ± 0.018; Tables S17 and S18). Nucleotide diversity was low (π = 0.648 × 10^−4^), as expected for the non-recombining Y chromosome. Phylogenetic reconstruction and network analysis revealed that Honghe cattle comprised two major paternal lineages, Y3a (East Asian indicine) and Y3b (South Asian indicine), indicating an indicine-dominant paternal origin. In addition, one bull was assigned to the Y1 lineage (European taurine) and two bulls to the Y2a lineage (Eurasian taurine) (Figure 5 and Figure S15). The lineage proportions were Y1 (4.5%), Y2a (9.1%), Y2b (0%), Y3a (50.0%) and Y3b (36.4%), with the two Y3 sub-lineages together accounting for 86.4% of Honghe bulls.

## 4. Discussion

Understanding the genetic structure of indigenous livestock breeds is essential for biodiversity conservation and sustainable breeding, particularly under accelerating climate pressures [39,40]. As a newly recognized breed facing considerable conservation challenges, Honghe cattle have lacked genomic characterization. To address this gap, we performed a genomic analysis using the largest whole-genome variant dataset assembled for this breed to date. Previous studies have shown a north-to-south gradient in Yunnan local cattle, with taurine ancestry decreasing and indicine ancestry increasing toward the south [10].

Our ancestral composition analysis not only confirmed this pattern but also quantified the ancestral proportions in Honghe cattle. We selected reference populations with established breed backgrounds: 25 South Asian indicine, 21 East Asian indicine, 21 East Asian taurine, and 28 European taurine individuals, together with 32 individuals from two Yunnan local breeds (Dianzhong and Deng Chuan) geographically close to Honghe cattle, as reference populations. This analysis revealed that Honghe cattle comprise 54.7% East Asian indicine, 26.5% South Asian indicine, and 18.8% taurine ancestry. The taurine ancestry likely reflects historical introduction of European taurine breeds and crossbreeding with local cattle for genetic improvement, which may have led to the loss of some indigenous characteristics. In the paternal lineage analysis, Honghe cattle were derived predominantly from Y3a and Y3b, with a small proportion from Y2 and Y1, further supporting the admixed genetic background.

PCA further corroborated the population structure inferred above: although the first three principal components cumulatively explained only 13.3% of the total variance, one-way ANOVA showed that PC1–PC3 each accounted for more than 94% of the among-population variance along their respective axes, confirming that the low variance proportions do not compromise the reliability of the population structure inference. Consistent with the admixed origin revealed by ADMIXTURE, Honghe cattle were positioned between the taurine and indicine clusters along PC1. Furthermore, we found that Honghe cattle are genetically closely related to Dianzhong cattle, with an *F*_ST_ value of only 0.008 between the two breeds, and the two grouped consistently together in both hierarchical clustering and neighbor-joining analyses. This likely reflects their geographical proximity and the absence of major geographic barriers, which facilitated a shared genetic background or frequent gene flow between the two populations. Nevertheless, as a newly recognized local breed, Honghe cattle retain unique genetic characteristics—most notably a markedly different demographic history (Figure S7, trajectories based on small sample sizes have wide confidence intervals and require cautious interpretation), the lowest inbreeding level among the Chinese breeds (F_ROH_ = 0.032), and a relatively high number of breed-specific SNPs (78.4 k per individual on average).

The nucleotide diversity of Honghe cattle (π = 0.00327) was lower than that of East Asian indicine but higher than all other comparative breeds. Previous studies have attributed the high genetic diversity of Chinese indicine cattle to introgression from *Bos javanicus* (banteng) [8,9]. As an admixed lineage, Honghe cattle have inherited substantial genomic ancestry from East Asian indicine, which may explain their relatively high diversity. Inbreeding analysis further showed that Honghe cattle rank among the least inbred populations (mean F_ROH_ = 0.032 ± 0.003; median = 0.025), exceeding only South Asian indicine—consistent with the nucleotide diversity results and indicating considerable potential for selective breeding. In the ROH analysis, European commercial breeds (European taurine and East Asian taurine) exhibited the highest numbers and total lengths of long ROH segments (>4 Mb), consistent with previous findings and attributable to intensive commercial breeding practices [14,41,42]. In contrast, Honghe cattle were dominated by short segments (0.5–1 Mb), with intermediate-to-low counts and lengths of long ROHs, indicating limited recent inbreeding accumulation. This feature may be associated with the long-term open or semi-open management of Honghe cattle as a Yunnan local breed, which likely facilitated gene flow with neighboring populations, thereby limiting inbreeding accumulation and maintaining high genetic diversity.

Genes within long ROH segments (> 4 Mb, *n* = 96) were enriched in metabolic and energy-balance pathways—including metabolic pathways, insulin resistance and MAPK signaling—patterns consistent with selection for nutrient utilization and thermoregulatory homeostasis under subtropical conditions. More strikingly, the strongest ROH island harbored a proteostasis gene cluster spanning the three subcellular protein-quality-control compartments (*HSPA9*/mitochondria [43], *SIL1*/ER [44], *DNAJC18*/cytosol), with GO enrichment of “response to unfolded protein” providing independent support. Heat stress is a major constraint on livestock production in tropical and subtropical regions [45], and *DNAJC18* has been repeatedly implicated in temperature adaptation in cattle [46,47,48]. *HSPA9* and *SIL1* have also been identified as candidate genes in heat-adapted cattle populations [47,49]. The complementary expression of *HSPA9* in gravid endometrium and *DNAJC18* in the oviduct further suggests that this island may be involved in reproductive robustness under heat stress. In addition, the GO term DNA replication origin binding and candidate genes in other islands (*MYL4* and *SGK3*) implicate the ROH islands in production traits including heart and coat characteristics [50,51]. Overall, the ROH landscape of Honghe cattle reflects mild recent inbreeding together with genomic regions potentially associated with environmental adaptation and production performance, warranting functional validation.

It should be noted that our selection-signature analyses may be affected by population structure and demographic history. Honghe cattle carry 81.2% indicine ancestry, and the studied breeds differ markedly in their bottleneck histories: European taurine and East Asian taurine breeds experienced severe bottlenecks, whereas the Yunnan breeds exhibit distinct demographic trajectories. Such heterogeneity can inflate haplotype-based statistics and generate false-positive signals. To mitigate this risk, *iHS* and CLR were computed within breeds only, and only regions convergently supported by at least two complementary statistics were retained.

We used *iHS* and CLR to jointly identify 95 candidate genes, among which *CYP19A1*, *USH2A*, *SLC35B1* and *ACAD11* exhibited relatively strong selection signals. *CYP19A1* encodes cytochrome P450 aromatase, which is responsible for the conversion of androgen precursors to estradiol [52]. *CYP19A1* may contribute to reproductive performance in female Honghe cattle by regulating estrogen synthesis and secretion, thereby influencing follicular cell growth [53,54]. *SLC35B1*, a key regulator of lactose synthesis, may influence the reproductive potential of Honghe cows [55,56], while *USH2A* has been reported as a selection target in several other cattle breeds [57,58,59]. *ACAD11*, previously linked to feed intake efficiency in Sahiwal and dairy cattle [60,61,62], may contribute to the roughage tolerance of Honghe cattle.

In addition, cross-population selection comparisons using *F*_ST_ and XP-EHH revealed significant genomic differentiation between Honghe cattle and East Asian taurine cattle. A total of 443 candidate genes were identified, among which *MCM6*, *NAPEPLD* and *HMGA2* exhibited particularly pronounced differentiation signals. *MCM6* is a DNA replication regulatory factor that, together with other MCM family proteins, forms a hexameric complex around DNA and play a key role in cell cycle maintenance [63]. Moreover, haplotypes of the *MCM6-LCT* region have been shown to be associated with lactose metabolism, suggesting that this gene may be related to milk production traits in Honghe cattle [64,65]. It should be noted that *MCM6-LCT* is a classic taurine locus, and the observed differentiation signal may partly reflect introgression of taurine ancestry rather than selection within indicine cattle. *NAPEPLD* encodes N-acyl phosphatidylethanolamine phospholipase D, a key enzyme in the synthesis of endogenous cannabinoids. Studies have shown that this enzyme interacts with gut microbiota to influence fat deposition and can also affect short-term feeding behavior through the gut–brain axis, indicating a role in lipid metabolism [66], that may be relevant to meat quality traits in Honghe cattle.

Notably, *HMGA2* exhibited a distinctive haplotype pattern together with pronounced differentiation signals, suggesting that it may have been subjected to strong selection in Honghe cattle. *HMGA2* encodes a structural transcription factor that binds AT-rich DNA sequences and induces conformational changes affecting transcription [67]. Deficiency of this gene has been shown to cause dwarfism in mice and rabbits, making it a strong candidate gene for body size [68]. Moreover, similar findings have been reported in small-sized yak and Tibetan cattle [69]. Given that Honghe cattle are the smallest-bodied local breed in Yunnan Province, this selection signal likely shaped their small body size, a trait that reduces metabolic costs and facilitates adaptation to the tropical environment.

Nevertheless, several limitations of this study should be acknowledged: (1) the sample size was relatively modest, and the analysis relied primarily on SNP data without systematic evaluation of other variant types such as structural variations; (2) because all reads were mapped to the taurine reference assembly (ARS-UCD1.2), reference bias in diverged genomic regions may have led to underestimation of heterozygosity; (3) owing to limited tissue accessibility and the absence of independent Honghe cattle genotype data, the candidate genes have not been experimentally validated, and the relevant findings should be regarded as hypothesis-generating. However, our main conclusions remain robust for the following reasons. First, the population genetic parameters estimated in this study are consistent with those reported for other Chinese indigenous cattle breeds using larger sample sizes. Second, the reference bias acts uniformly across indicine-derived breeds, and ROH detection in poorly mappable regions is limited by SNP-density criteria, so that our among-breed comparisons and F_ROH_ estimates are conservative rather than inflated. Third, the clear ancestry patterns and the convergent selection signals detected by multiple methods (CLR, *iHS*, and XP-EHH) are unlikely to be artifacts of limited sample size, as strong selection signals remain detectable even at moderate sample sizes. Future studies incorporating three-population branch statistics and pairwise comparisons with closely related indicine breeds to disentangle lineage divergence from Honghe-specific selection, together with tissue-specific expression assays and association testing in independent populations, will further consolidate these findings and facilitate their application in the conservation and genetic improvement of Honghe cattle.

This study provides a genomic characterization of Honghe cattle, revealing a predominantly indicine-derived admixed origin, relatively high genetic diversity, and selection signatures linked to environmental adaptation. We identified candidate genes associated with immunity, meat quality, milk production and body size. These findings may provide a scientific basis for breeding strategies and climate resilience in Honghe cattle. Future studies should integrate multi-omics approaches and gene editing for functional validation of the candidate genes, and combine these with phenotypic data to more comprehensively elucidate the molecular mechanisms underlying environmental adaptation. This would provide a stronger foundation for germplasm conservation and molecular breeding of this breed.

## 5. Conclusions

In this study, we characterized the genomic variation landscape of Honghe cattle, a newly recognized breed, using whole-genome resequencing data. Our investigation of population structure, genomic diversity, and paternal genetic lineages will provide a foundation for the genetic evaluation of this breed. In parallel, we identified a series of potential candidate genes that may influence meat quality, body size and growth, disease resistance, and heat tolerance, offering directions for the scientific breeding of this breed. Furthermore, these findings will enrich the genetic resource database of Yunnan local breeds and serve as a valuable reference for future studies on indigenous cattle in Yunnan Province.

## Data Availability

All raw sequencing data have been submitted to the NGDC (https://ngdc.cncb.ac.cn, accessed on 5 November 2026) under the BioProject accession number PRJCA072462.

## Supplementary Materials

The following supporting information can be downloaded at https://www.mdpi.com/article/10.3390/ani16213541/s1. Figure S1: Cross-validation (CV) errors for ADMIXTURE ancestry models with *K* ranging from 2 to 6; Figure S2: Principal component analysis of cattle with PC1 against PC3; Figure S3: Principal component analysis of cattle with PC2 against PC3; Figure S4: Scree plot of the first ten principal components from the PCA of 189 individuals. Blue line: proportion of variance explained by each individual PC (left y-axis); orange line: cumulative proportion of variance explained (right y-axis); Figure S5: Average length of three types of ROH in each population; Figure S6: KEGG enrichment analysis of long ROH in Honghe cattle (Length > 4 Mb); Figure S7: Demographic history of cattle populations inferred by SMC++; Figure S8: Haplotype heatmap of the *SIL1* gene (7: 50.26 Mb–50.52 Mb); Figure S9: Haplotype heatmap of the *MATR3* gene (7: 50.59 Mb–50.62 Mb); Figure S10: Haplotype heatmap of the *CTNNA1* gene (7: 49.91 Mb–50.26 Mb); Figure S11: Haplotype heatmap of the *LRRTM2* gene (7: 50.190 Mb–50.194 Mb); Figure S12: Haplotype heatmap of the *PAIP2* gene (7: 50.63 Mb–50.65 Mb); Figure S13: Venn diagram of candidate genes identified by CLR and *iHS* methods; Figure S14: Venn diagram of candidate genes identified by *F*_ST_ and XP-EHH methods; Figure S15: Maximum Likelihood (ML) phylogenetic tree of Y-chromosomal haplotypes. Table S1: Summary of sequencing data; Table S2: Distribution of SNPs identified in Honghe cattle within various genomic regions annotated by ANNOVAR; Table S3: Eigenvalues and variance contribution of the first ten principal components; Table S4: Pairwise *F*_ST_ among populations; Table S5: Summary of SNP numbers across populations; Table S6: Count and length of three ROH types across populations; Table S7: Information of 73 long ROH (Length ≥ 4 Mb) fragments in Honghe cattle; Table S8: KEGG enrichment analysis of genes annotated in long ROH fragments of Honghe cattle (Corrected *p*-Value < 0.05); Table S9: Summary of genes in top 1% ROH islands of Honghe cattle; Table S10: KEGG pathway analysis of genes in ROH hotspot regions of Honghe cattle (Corrected *p*-Value < 0.1); Table S11: GO enrichment of genes in ROH hotspot regions of Honghe cattle (Corrected *p*-Value < 0.05); Table S12: A summary of genes from CLR in Honghe cattle; Table S13: A summary of genes from iHS in Honghe cattle; Table S14: A summary of genes from *F*_ST_; Table S15: A summary of genes from XP-EHH; Table S16: KEGG enrichment analysis of genes overlapping *F*_ST_ and XP-EHH signals in Honghe cattle (Corrected *p*-Value < 0.05); Table S17: Individual information used for Y-chromosome analysis (dark blue indicates reference individuals); Table S18: Information of haplotypes in Honghe cattle.

## Abbreviations

The following abbreviations are used in this manuscript:

SNP	Single nucleotide polymorphisms
ROH	Runs of homozygosity
CLR	Composite likelihood ratio test
*F*_ST_	Fixation index
*iHS*	Integrated haplotype score
XP-EHH	Cross population extended haplotype homozygosity
PCA	Principal component analysis
NJ	Neighbor-Joining
ML	Maximum Likelihood
KEGG	Kyoto Encyclopedia of Genes and Genomes
GO	Gene Ontology
