# Supplementary material for: Whole-Genome Resequencing Reveals Genetic Diversity and Selection Signatures in Honghe Cattle
Source: Animals (Basel). 2026 Nov 9;16(21):3541. doi: 10.3390/ani16213541 (PMC13761873; doi:10.3390/ani16213541)
Supplement: Supplementary file 1 [file animals-16-03541-s001.zip › Supplementary Figure S1-S15.pdf]

Article

Whole-Genome Resequencing Reveals Genetic Diversity and Selection Signatures in Honghe Cattle.

Supplementary Figures:

Figure S1. Cross-validation (CV) errors for ADMIXTURE ancestry models with K ranging from 2 to 6.

Figure S2. Principal component analysis of cattle with PC1 against PC3.

Figure S3. Principal component analysis of cattle with PC2 against PC3.

Figure S4. Scree plot of the first ten principal components from the PCA of 189 individuals. Blue line: proportion of variance explained by each individual PC (left y-axis); orange line: cumulative proportion of variance explained (right y-axis).

Figure S5. Average length of four types of ROH in each population.

Figure S6. KEGG enrichment analysis of long ROH in Honghe cattle (Length > 4Mb).

Figure S7. Demographic history of cattle populations inferred by SMC++.

Figure S8. Haplotype heatmap of the *SIL1* gene(7: 50.26Mb ~ 50.52Mb).

Figure S9. Haplotype heatmap of the *MATR3* gene(7: 50.59 Mb ~ 50.62Mb).

Figure S10. Haplotype heatmap of the *CTNNA1* gene(7: 49.91Mb ~ 50.26Mb).

Figure S11. Haplotype heatmap of the *LRRTM2* gene(7: 50.190Mb ~ 50.194Mb).

Figure S12. Haplotype heatmap of the *PAIP2* gene(7: 50.63Mb ~ 50.65Mb).

Figure S13. Venn diagram of candidate genes identified by CLR and *iHS* methods.

Figure S14. Venn diagram of candidate genes identified by F_{ST} and XP-EHH methods.

Figure S15. Maximum Likelihood (ML) phylogenetic tree of Y-chromosomal haplotypes.

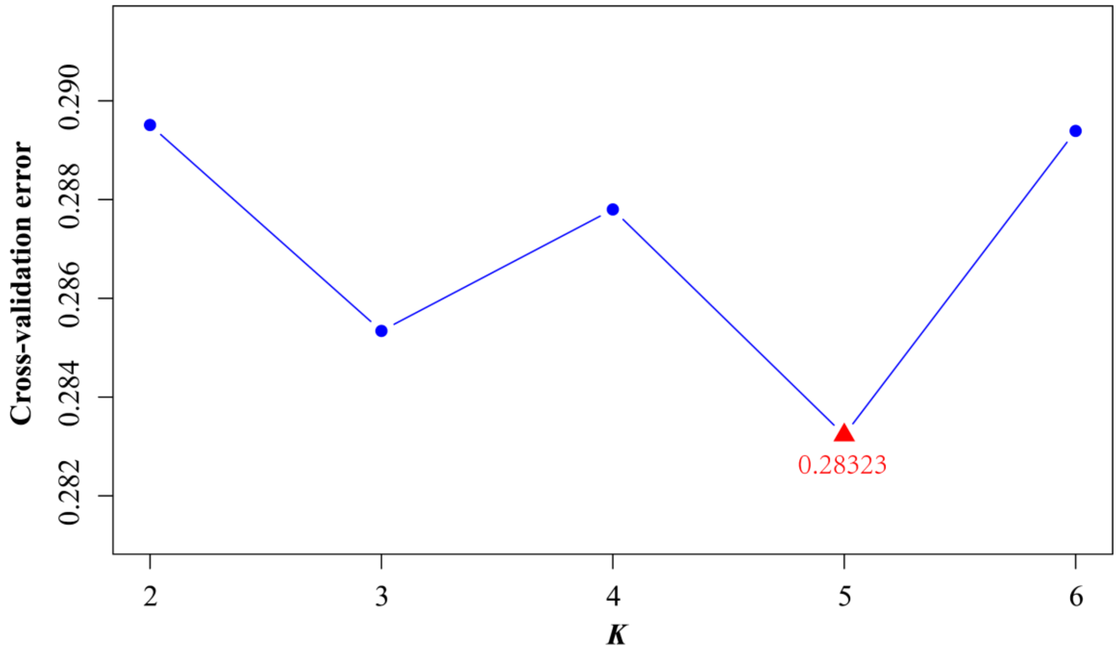

Figure S1. Cross-validation (CV) errors for ADMIXTURE ancestry models with *K* ranging from 2 to 6. The red triangle indicates the lowest point, which represents the optimal clustering.

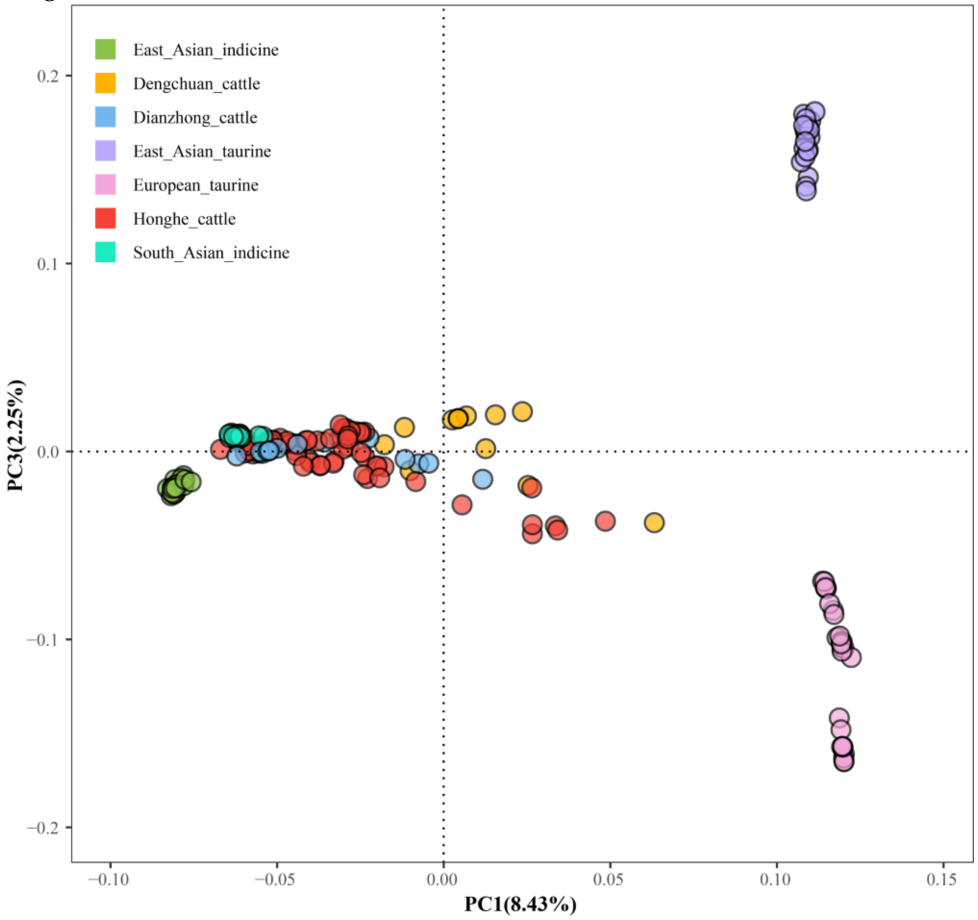

Figure S2. Principal component analysis of cattle with PC1 against PC3.

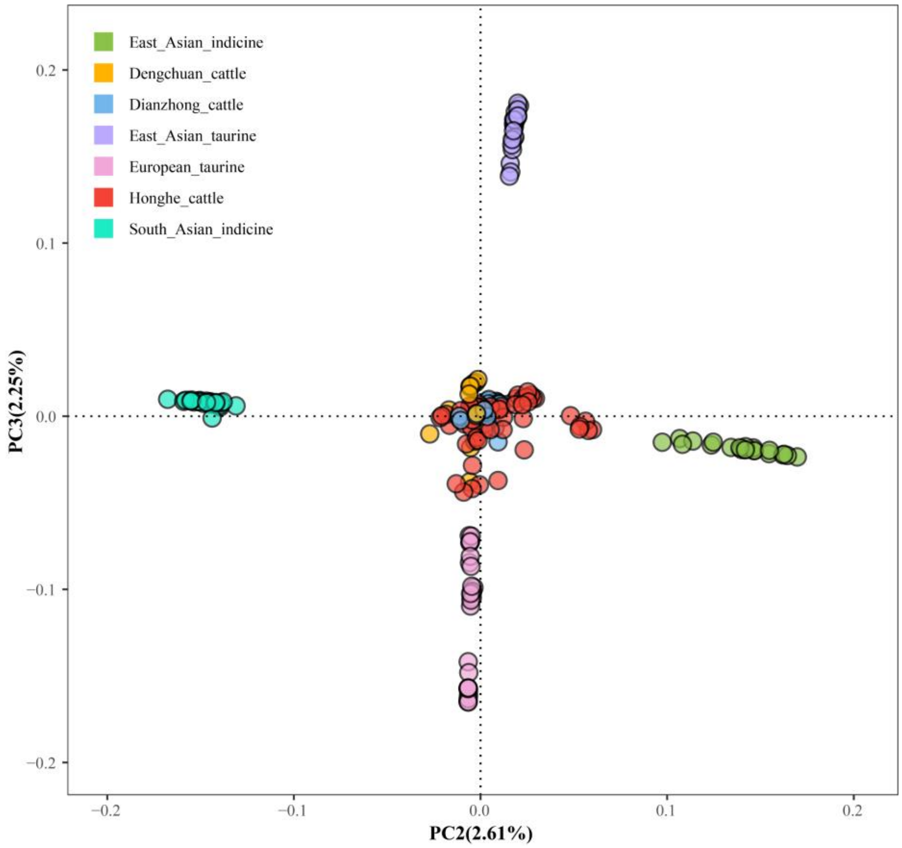

Figure S3. Principal component analysis of cattle with PC2 against PC3.

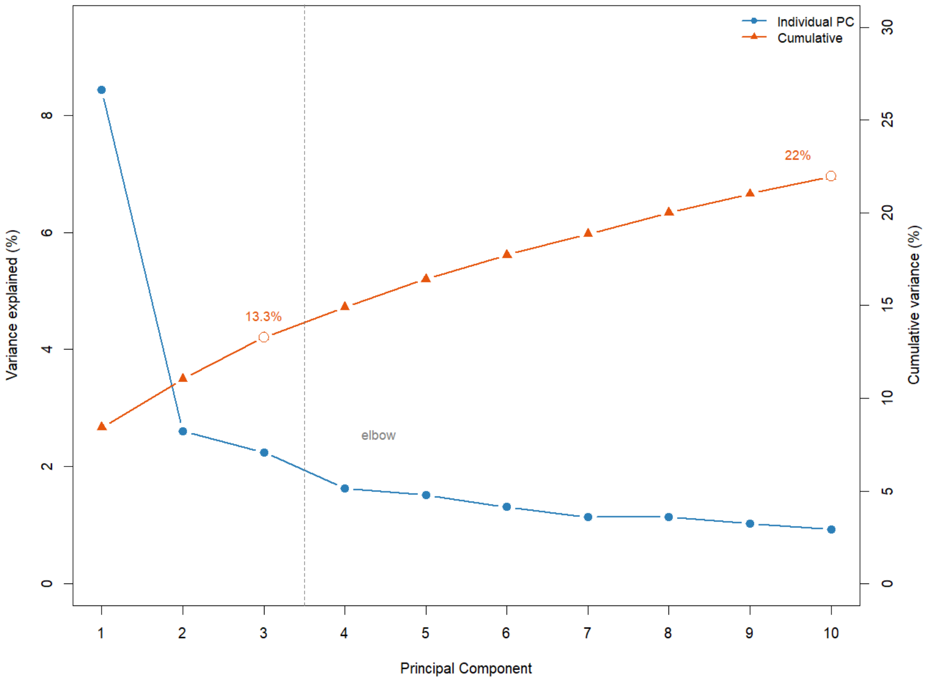

Figure S4. Scree plot of the first ten principal components from the PCA of 189 individuals. Blue line: proportion of variance explained by each individual PC (left y-axis); orange line: cumulative proportion of variance explained (right y-axis).

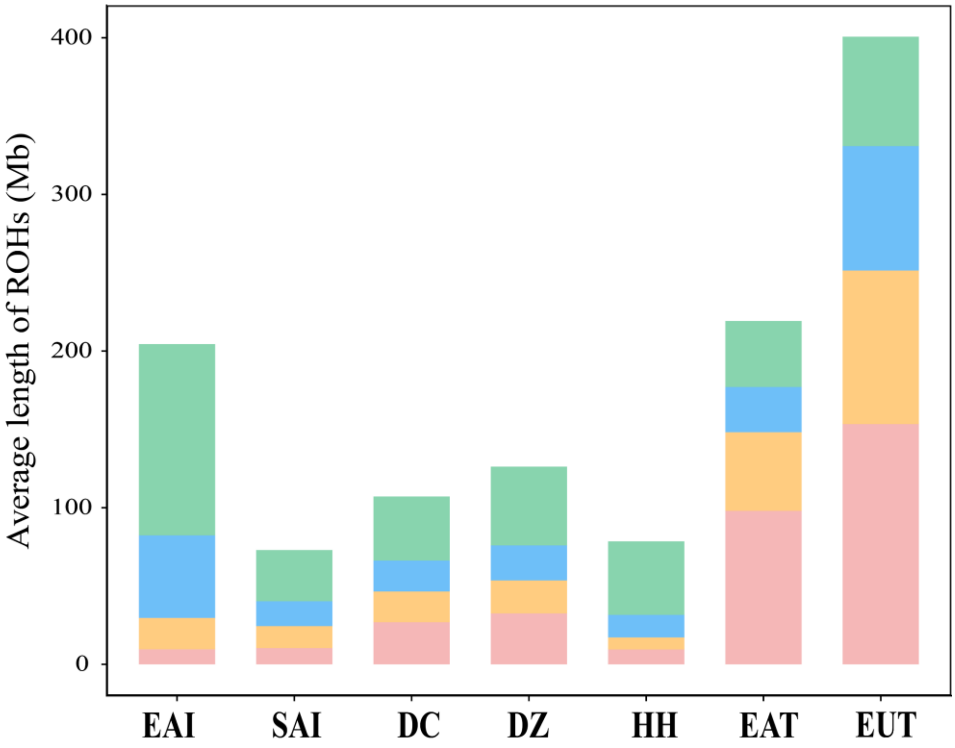

Figure S5. Average length of four types of ROH in each population.

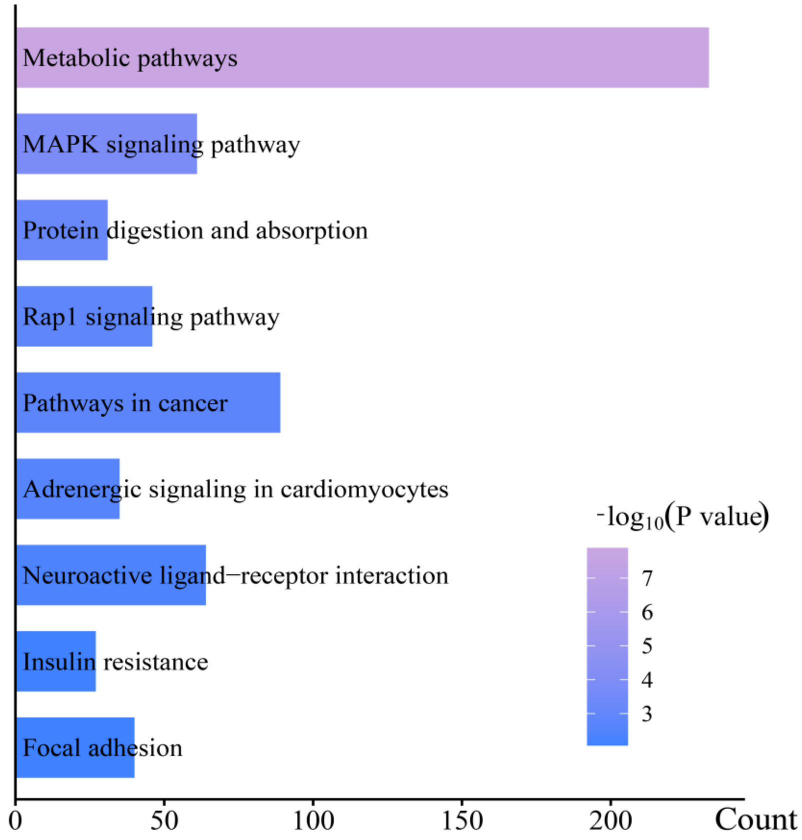

Figure S6. KEGG enrichment analysis of long ROH in Honghe cattle (Length > 4 Mb).

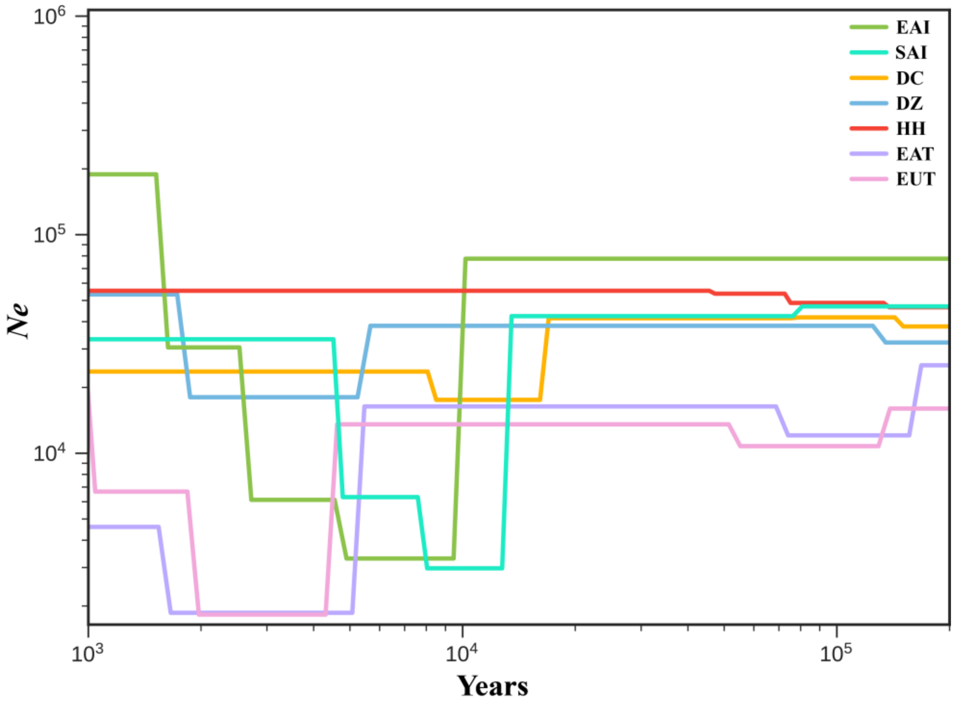

Figure S7. Demographic history of cattle populations inferred by SMC++.

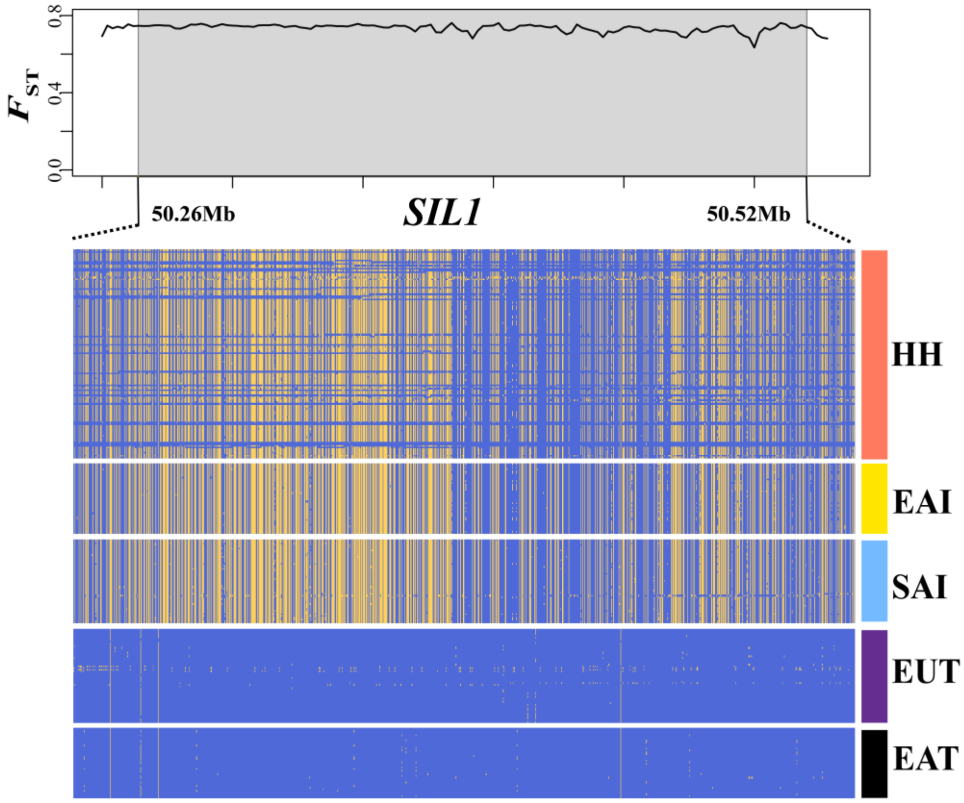

Figure S8. Haplotype heatmap of the *SIL1* gene(7: 50.26Mb ~ 50.52Mb).

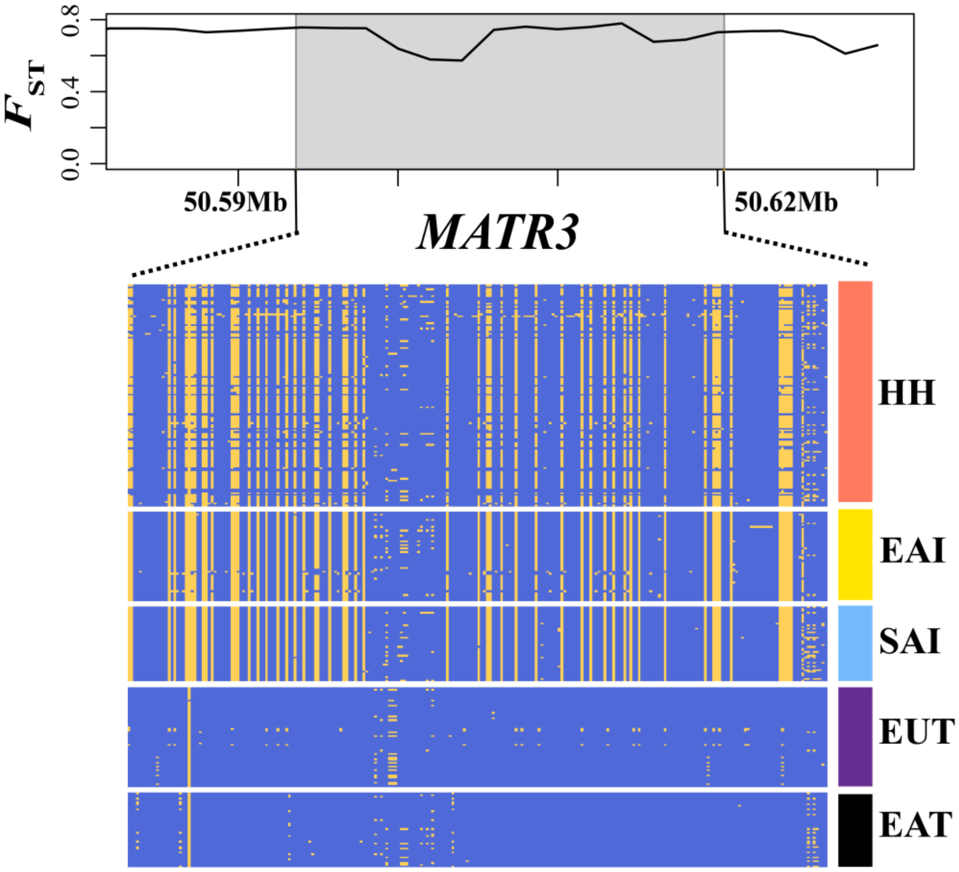

Figure S9. Haplotype heatmap of the *MATR3* gene(7: 50.59 Mb ~ 50.62Mb).

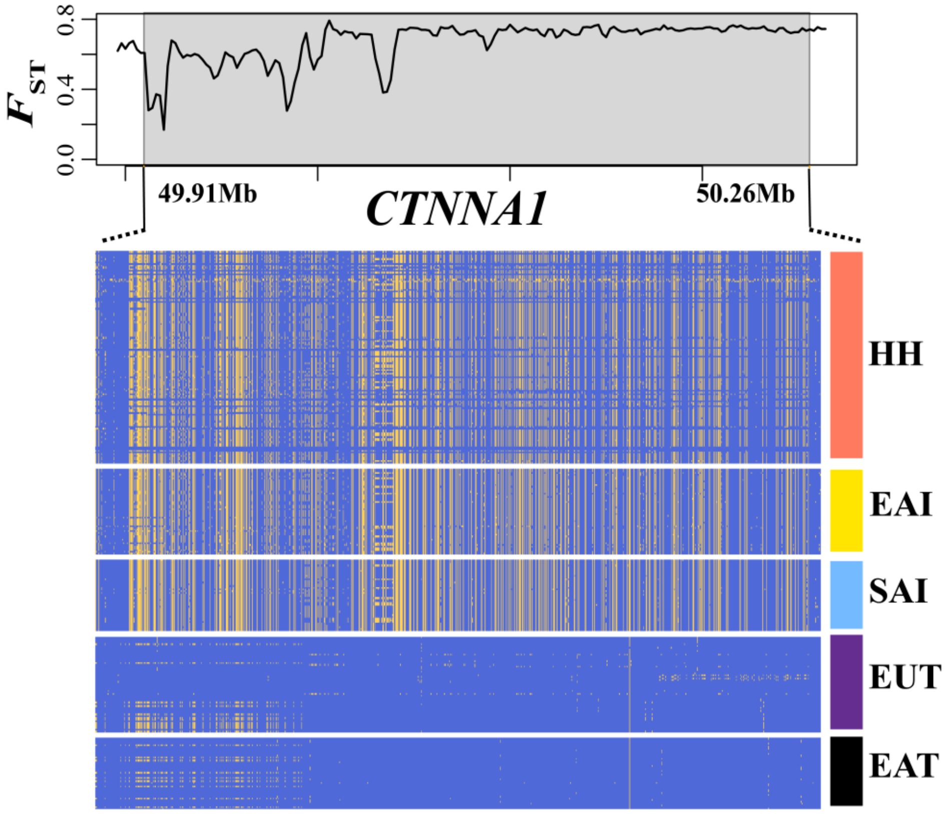

Figure S10. Haplotype heatmap of the *CTNNA1* gene(7: 49.91Mb ~ 50.26Mb).

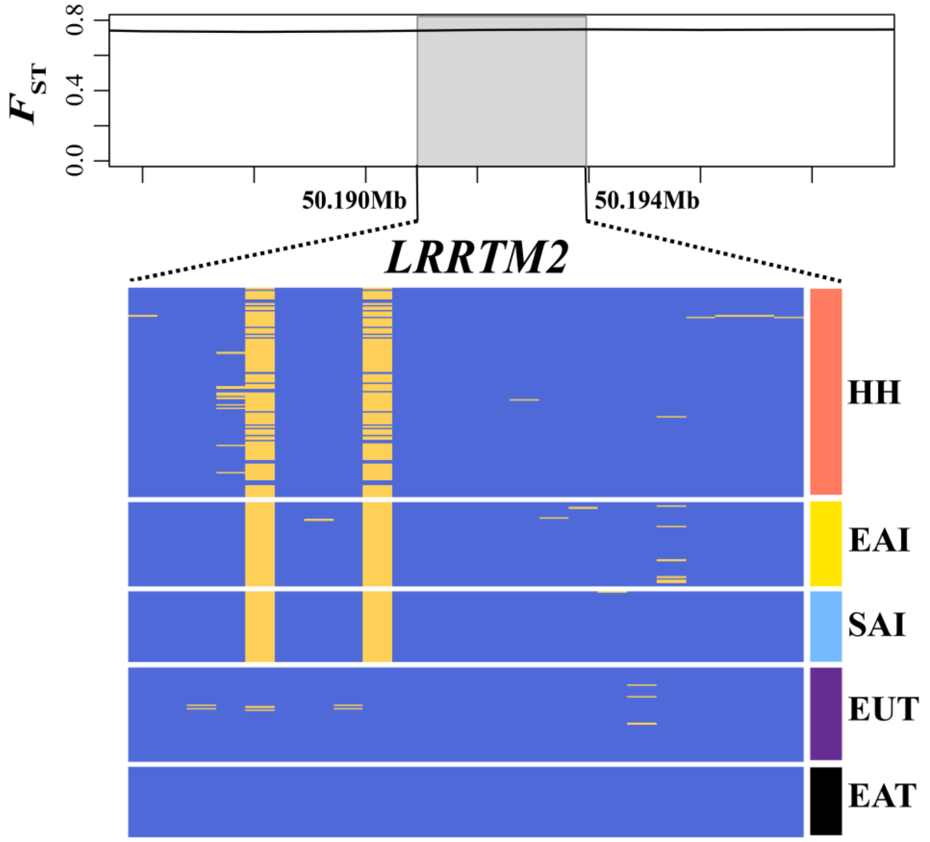

Figure S11. Haplotype heatmap of the *LRRTM2* gene(7: 50.190Mb ~ 50.194Mb).

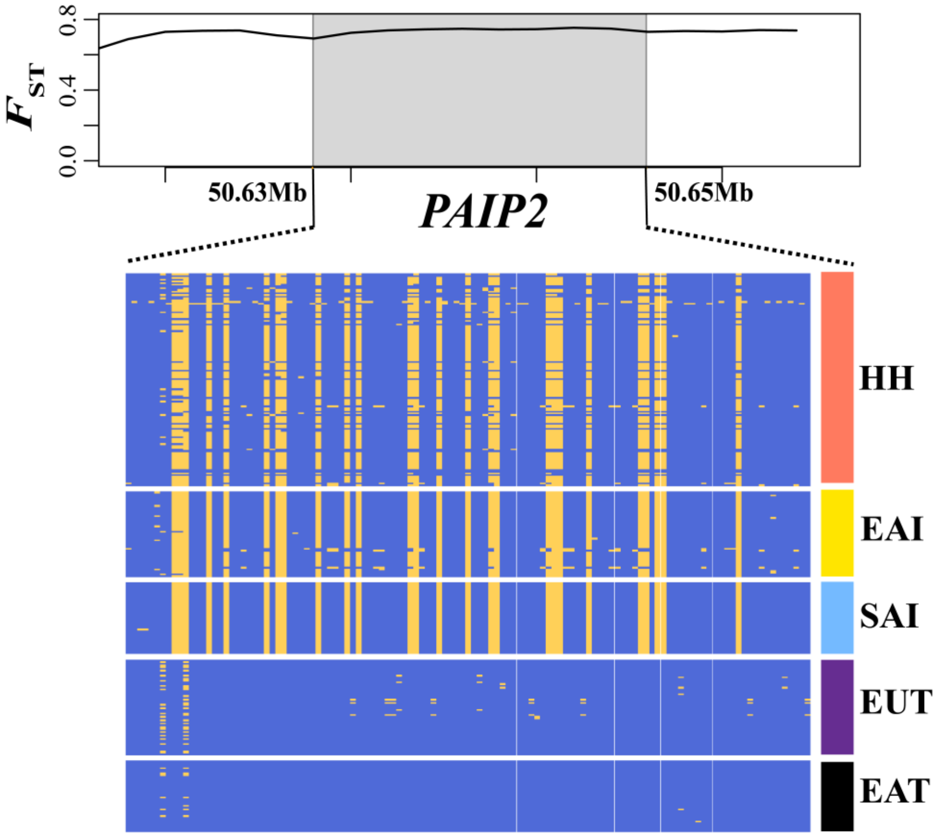

Figure S12. Haplotype heatmap of the *PAIP2* gene(7: 50.63Mb ~ 50.65Mb).

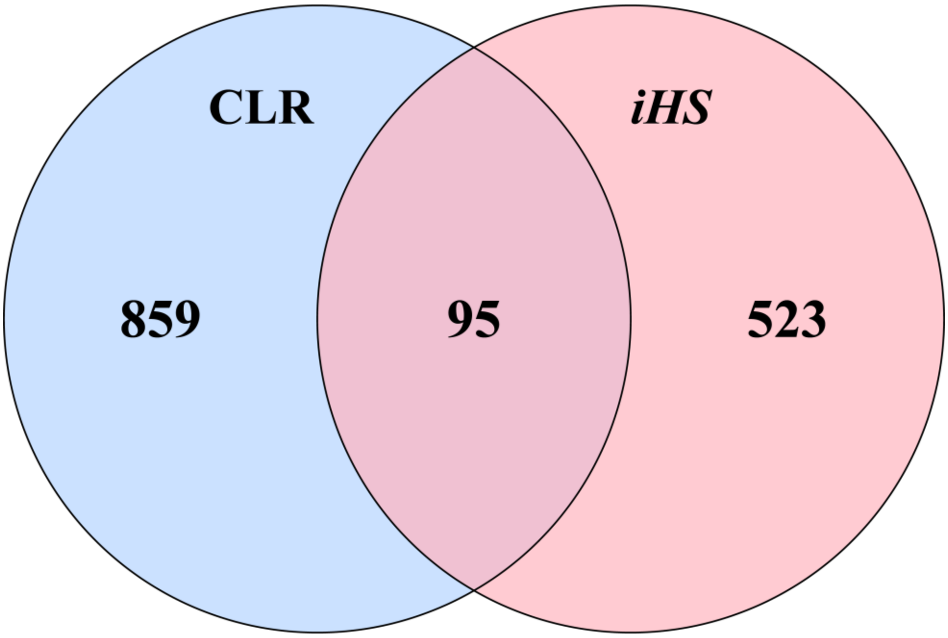

Figure S13. Venn diagram of candidate genes identified by CLR and *iHS* methods.

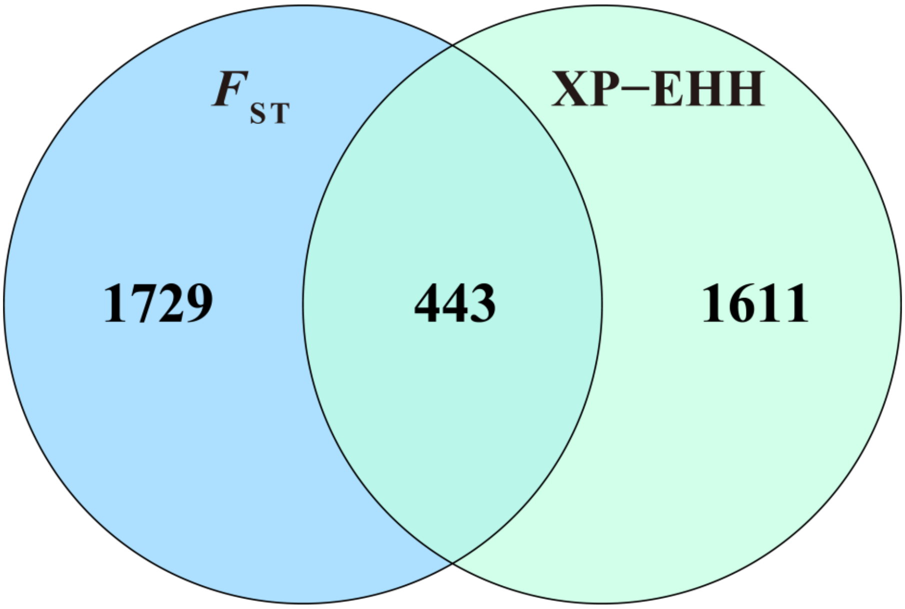

Figure S14. Venn diagram of candidate genes identified by F_{ST} and XP-EHH methods.

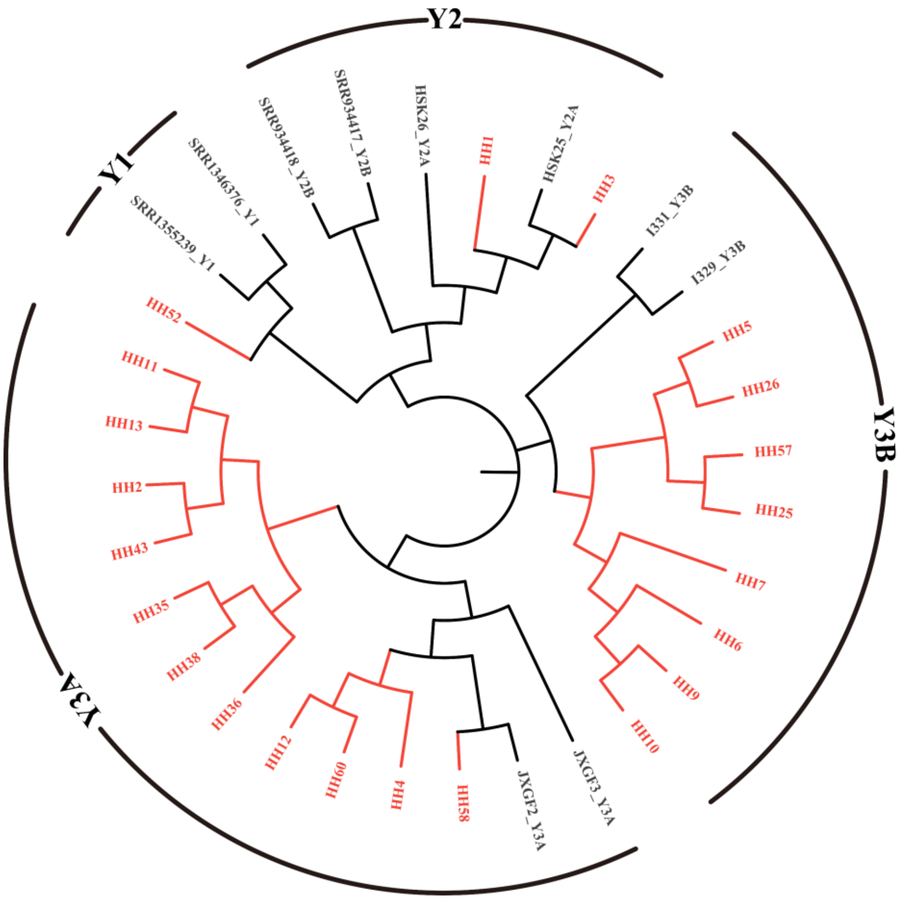

Figure S15. Maximum likelihood (ML) phylogenetic tree of Y-chromosomal haplotypes. The target population is shown in red, while the reference population is shown in black.
